# Environmental Risk Factors Associated with Child Stunting: A Systematic Review of the Literature

**DOI:** 10.29024/aogh.2361

**Published:** 2018-11-05

**Authors:** Dwan Vilcins, Peter D. Sly, Paul Jagals

**Affiliations:** 1 Child Health Research Centre, The University of Queensland, Center for Children’s Health Research South Brisbane, Queensland, AU; 2 School of Public Health, University of Queensland, Brisbane, Queensland, AU

## Abstract

**Background:**

Stunting, a form of malnutrition characterized by impaired linear growth in the first two years of life, affects one quarter of children globally. While nutritional status remains the key cause of stunting, there is evidence that environmental risk factors are associated with stunting.

**Objective:**

The objective of this review is to explore the current literature and compile the environmental risk factors that have been associated with stunting. Further, we seek to discover which risk factors act independently of nutritional intake.

**Methods:**

A systematic search of the literature was performed using PubMed, EMBASE, Scopus, TOXNET, and CINAHL. A search of the grey literature was conducted. Papers were included in this review if they examined an association between childhood stunting and exposure to environmental risk factors.

**Findings:**

We included 71 reports in the final analysis. The included studies showed that foodborne mycotoxins, a lack of adequate sanitation, dirt floors in the home, poor quality cooking fuels, and inadequate local waste disposal are associated with an increased risk of childhood stunting. Access to safe water sources was studied in a large number of studies, but the results remain inconclusive due to inconsistent study findings. Limited studies were available for arsenic, mercury, and environmental tobacco, and thus their role in stunting remains inconclusive. The identified research did not control for nutritional intake. A causal model identified solid fuel use and foodborne mycotoxins as being environmental risk factors with the potential to have direct effects on childhood growth.

**Conclusions:**

A diverse range of environmental risk factors are, to varying degrees, associated with stunting, demonstrating the importance of considering how the environment interacts with nutrition. Health promotion activities may be more effective if they consider environmental factors alongside nutritional interventions.

## Introduction

It is estimated that one in four children under the age of five years are failing to grow along the optimum trajectory set out in the World Health Organization’s Child Growth Standards [1]. This failure to grow is known as stunting, a term given to impaired linear growth (length/height for age) in the early years of life, which results in failure to reach a height by adulthood implied by genetic potential [1,2,3]. Stunting is a manifestation of malnutrition and is a significant health problem. Global predictions indicate that one in five children will be stunted in 2020 [4]. Stunting can result in negative health effects across the lifespan, such as life-threatening complications during birthing, reduced cognitive performance and development, poorer school attendance, and reduced adult earning capacity [2,3,5,6]. Reports have also linked sub-optimal body composition and non-communicable chronic disease risk factors in adults, such as a pre-disposition to obesity, high blood pressure, and harmful lipid profiles, to childhood stunting [5,6]. Further, it is anticipated that climate change will cause significantly more stunting through a reduction of food security [7].

The key window for stunting is from conception up to two years of age, commonly referred to as the first 1,000 days [1,2]. During this time an affected child is considered to be in a process of growth failure, or stunting. After the age of two years the rate of growth slows down, and the child is considered stunted [8]. The indicator for stunting is height/length compared to a healthy reference standard; a child more than two standard deviations below the median height-for-age is considered stunted [1,3,4].

Historically, research into stunting has focused on dietary intake, yet a growing body of evidence has shown an important role for the natural and physical environment in child health. Interactions between environment and nutrition present an interesting dynamic, where an interplay between environmental factors and nutritional status may lead to changes in health status. An example of this is iron deficiency, which potentially leads to increased lead absorption [9], or infection with parasites, which is associated with stunting [10,11,12]. We hypothesize that some environmental agents work independently of nutrition to negatively affect child growth. We searched for previous reviews and identified three systematic reviews exploring environmental risk factors and stunting. While useful, one review was geographically limited to sub-Saharan Africa [13], whilst the remaining reviews did not discuss specific environmental risk factors that could be associated with stunting [14,15]. The following questions were thus posed for this review: which environmental risk factors are associated with childhood stunted growth, and which risk factors have an effect on child growth that is independent of nutritional intake?

## Methodology

A systematic literature search was conducted to identify environmental risk factors linked to childhood stunting. The search strategy is reported below. This review takes a broad approach to environmental search terms, to allow for a range of risk factors to be identified.

### Literature search strategy

A review protocol was developed following the *Preferred reporting items for systematic review and meta-analysis protocols* (PRISMA-P) 2015 [16], and the search conducted for science reports on PubMed, EMBASE, Scopus, TOXNET, and CINAHL (EBSCO host interface) from their inception until 1 June 2015. A ‘grey’ literature search was conducted using Open Grey, The World Health Organization website, the World Health Organization library online catalogue (WHOLIS), UNICEF library, Open Access Thesis and Dissertation, Google, World Bank eLibrary, and OECD iLibrary. Finally, the reference lists of reviewed studies were scanned to detect studies missed by the initial search.

The search terms used in combinations were ‘stunting’ (Stunt*), ‘growth’ (body size, height, body height, child development), and ‘environment’ (environ*). Medical subject headings were used if the database allowed. The complete search strategy for PubMed is presented in Table 1.

**Table 1:** PubMed search terms.


(((“Growth and Development”[Mesh:NoExp] OR “Growth”[Mesh:NoExp] OR “Body Size”[Mesh:NoExp] OR (height*[tiab]) OR “Body Height”[Mesh])) AND stunt*) AND environ*


### Eligibility criteria

Research studies were selected for review if they a) used an outcome that measured stunting, b) examined exposure to a classical or emerging environmental risk factor, c) examined exposure occurring from conception up to the age of two years, and d) were published in the English language. This review primarily focused on risks that can be modified through activities such as environmental protection and health promotion. Works were excluded from this review if these are considered not sufficiently modifiable by interventions such as environmental health services; for instance, studies reporting on the role of topo-environmental factors such as altitude on child stunting, studies that examined nutritional risks (without an environmental component), and natural disasters. All included studies were appraised for risk of bias. The *Critical Appraisal Checklist* developed by the Centre for Occupational and Environmental Health at the University of Manchester [17] was used for observational studies, while experimental studies were assessed using the Cochrane Collaboration’s *Tool for Assessing Risk of Bias* [18]. Studies deemed as ‘definitely high’ risk of bias were excluded from this review. Data extraction and risk-of-bias assessment was performed by the first author, with a random sample assessed by the other authors.

### Strength of the evidence

After results synthesis, the hazards were grouped into the following arbitrary categories: ‘Strong evidence’ was used where the majority of studies that examined an environmental risk factor consistently reported an association with stunting. To meet this, a minimum of five studies on each factor was required, with greater than >70% of the available studies finding an association with stunting (positive or negative). ‘Inconclusive evidence’ was used for environmental risk factors if the included studies were divided in their consensus (less than 70% finding an association in the same direction) or too few in number (less than five) to conclusively find an association. Finally, ‘no association’ means that studies were included that examined an environmental risk factor, and they constituted a strong evidence base to suggest a lack of association between stunting and that risk factor (a minimum of five studies, with greater than >70% of these finding no association with child growth (positive or negative association).

A positive association with stunting was considered to be present if the hazard gave an adjusted odds ratio of more than one, with confidence intervals that did not cross the null. In the case of studies that compared groups, a positive association was considered present if the statistical tests reached significance.

## Results

### Outcomes of the literature search

**Figure 1:**
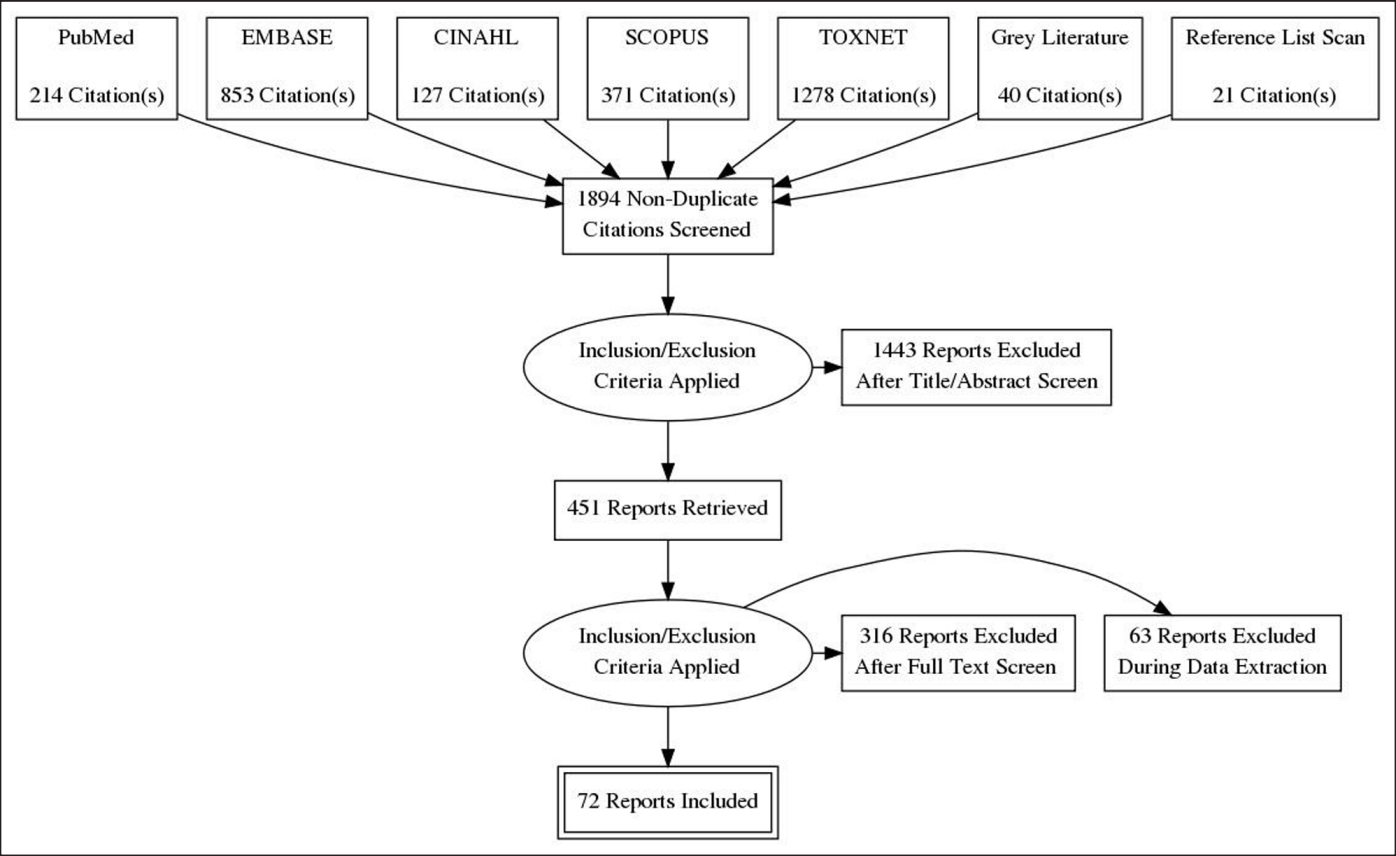
Flow of studies through the selection process.

The search yielded a total of 1,894 reports, after duplicates were removed. After these were screened applying the eligibility criteria, 71 reports were retained for review. Figure 1 illustrates this selection process. All of the studies used an observational study design, with the exception of two randomized controlled trials in the sanitation category. Table 2 presents the number of reports identified for each risk factor.

**Table 2:** Overview of the strength of the evidence of each environmental risk factor.

Category	Risk factor	No. of studies	Strength of the evidence

**Environmental settings**	Sanitation	34^#^	Strong*
	Safe drinking water – access	33	Inconclusive evidence*
	Arsenic in drinking water	2	Inconclusive evidence
	Environmental hygiene	3	Inconclusive evidence
	Solid waste disposal	10	Strong
	Building materials	13	Inconclusive evidence
	Flooring	5	Strong
	Animals	1	Inconclusive evidence
**Biological agents, and consequences**	Environmental enteropathy	3	Inconclusive evidence
	Intestinal parasites	10	Inconclusive evidence
	Malaria	2	Inconclusive evidence
	Mycotoxins	5	Strong
**Air quality**	Cooking fuels	7	Strong
	Environmental tobacco smoke	3	Inconclusive evidence
**Other hazards**	Pesticides	1	Inconclusive evidence
	Mercury in seafood	2	Inconclusive evidence
	Electromagnetic fields	1	Inconclusive evidence

* Description of these categories available in the methodology section. ^#^ Includes two experimental studies.

### Control for nutritional intake

As stated earlier, one of the primary objectives of this paper was to elucidate which environmental agents affect child growth, independent of nutritional intake. During data extraction, we realized that the included papers had not controlled for nutritional intake. This affected our aim of assessing environmental agents for effects on stunting, independent of nutrition. Thus this paper first presents an overview of the environmental risk factors that have been studied for an association with child growth, rating the strength of the evidence for each risk factor. We then present a directed graph to begin to explore which environmental agents may have an independent effect on child growth.

## Environmental risk factors associated with stunting

### Water, Sanitation, and Hygiene

Outside of nutrition interventions, the environmental health services that attracted the most attention for research into childhood stunting was water, sanitation, and hygiene (WASH). This is reflected in this review, with the bulk of reviewed reports focused on aspects of WASH.

#### Sanitation

Access to improved sanitation protected against stunting in the majority of reports (70.6% (24/34)) [19,20,21,22,23,24,25,26,27,28,29,30,31,32,33,34,35,36,37,38,39,40,41], with fewer (29.4% (10/34)) finding no such protective association [42,43,44,45,46,47,48,49,50,51]. This evidence suggests that access to proper sanitation protects against stunting in most settings. The included reports largely used a yes/no variable to assess personal latrine ownership; however, some studies included in this review show that personal latrine ownership may not be as important as the percentage of latrines at the village/neighborhood level. Two studies found that as the percentage of homes with access to a latrine increased, rates of stunting in the study areas (mostly villages) decreased [35,39]. Another study found that reducing the percentage of open defecators in a village was more effective in reducing stunting than increasing individual latrine ownership [37]. The study suggested that a measure of number of open defecators per square kilometre can account for 65% of the variation in children’s heights globally. It was also found that a 10% increase in open defecation leads to a 0.7% increase in the prevalence of stunting [38]. Lastly, residing in a village with a WASH program raised average heights by 0.3–0.4SD [26]. Even children who did not have access to a latrine but lived in the village experienced a similar increase in their height-for-age Z-score.

Another factor influencing the effect of sanitation on height-for-age Z-score is the appropriate use of latrines. One study found that the sanitation part of a WASH program did not result in improvements in height-for-age Z-score for children [44], attributing the results to an inadequate change in toileting habits as open defection still occurred despite having a new latrine.

#### Hygiene

Hygiene practices are known to be important to support child health, but most studies in this review did not examine the interactions of hygiene habits with access to a latrine. Two studies did examine hygiene practices as independent variables, and both found that improved hygiene practices (such as appropriate hand washing and presence of soap and water near latrine) was associated with reduced rates of stunting [33,52].

### Safe Drinking Water

#### Access

Access to safe water also featured prominently in the reviewed studies; however, whether it reduced child stunting was not clear. Just under half of the included papers (48% (16/33) found access to clean water sources reduced stunting [22,24,27,29,31,33,34,35,36,39,40,42,43,53,54,55], while 52% (17/33) of the studies found no protective association [19,20,21,23,28,30,32,37,45,46,47,48,50,51,56,57,58].

While the lack of consensus among these studies suggests that the protective effect of access to safe water on stunting cannot be confirmed, there are possible explanations for this: several of the papers combined access to water sources into a simple yes/no variable, with different sources categorized as non-improved (such as surface and rainwater catchment) [53] or improved (such as piped, well, and bottled) [35,55]. This simple dichotomous variable may mask the beneficial/detrimental effects of access to a single source. Further, there was limited regard of water storage and handling within the home. No included study tested the water for quality and safety, or commented on the seasonal effects on domestic water security. With these methodological limitations, it is difficult to infer the role of access to safe water in preventing stunting.

#### Arsenic in water

Some studies attempted to ascertain the role of waterborne arsenic in child growth. One study assessed exposure to arsenic by measuring urinary arsenic. This was associated with reductions in length that were significant for girls (but not boys) [59]. This contrasts with the findings of another reviewed study, which showed the percentage of methylarsonic acid in urine was positively associated with child height [60]. The authors hypothesized that rapid growth (as seen in childhood) creates a change in homocysteine levels which is commonly associated with elevated methylarsonic acid in urine, thus making it difficult to infer the role of arsenic exposure in child growth. The reviewed evidence was insufficient to conclude whether arsenic exposure is associated with child stunting.

### General environmental hygiene

Three papers examined what can be described as a general setting related to ‘environmental condition’. The environmental condition in this review is effectively defined by the hygiene of the domestic environment, and measured by combining environmental variables into a single measure [24,61].

One study used an environmental quality index, which combined scores for water source, sanitation, and hygiene into a single index figure [24]. They found that communities with a lower-quality index had higher stunting. Within those same lower-indexed communities were individual households that had higher incidence of stunting compared to households with higher-quality index scores.

The second study used a survey to rate environments as satisfactory or poor based on the responses on overcrowding and safe drinking water as well as sanitary waste disposal. This study found that poor environmental conditions were associated with stunting [52].

The remaining study rated a combination of water, sanitation, and hygiene factors to assess an environment as being clean or contaminated [61]. They found that stunting prevalence was 22% lower in the households rated as ‘clean’. The strength of this study was that the interviewer went to the house and visually inspected the services, compared with the previous two studies which relied of self-reporting of participants.

These papers provide some evidence to suggest that hygienic environmental settings are protective against child stunting, although we appear to still lack an understanding of the causal factors within unhygienic environments that mediate stunting.

### Solid waste disposal

The impact of solid waste disposal on stunting rates was examined in 10 papers. While two reports found no association between poor waste management and stunting [48,50], eight found that a lack of adequate waste removal from the community increased stunting rates [19,22,24,27,36,39,53,57,48,50]. These papers provide good evidence that solid waste products remaining in domestic environments is associated with childhood stunting.

### Housing

The material that a house was built from, including its flooring, were examined in several studies. Half of the papers (4/8) reported that poorer-quality housing materials were associated with stunting [20,24,36,53], while the other half reported that it was not [45,46,54,56]. Flooring type was shown to be important, with all the studies that examined flooring showing dirt floors were associated with stunting (100% (5/5)) [24,32,34,36,55].

The results of these studies provide some evidence that children living in homes with dirt floors are at an increased risk of being stunted. The hazards present in dirt floors that are causative of stunting cannot be elucidated in this review. For example, two of the papers originate from South America, where Chagas disease is endemic and may be the causative agent for stunting. Without further investigation into the hazards present in dirt flooring, we can only surmise that dirt floors are a risk factor for stunting. The association between stunting and building materials is inconclusive, due to the inconsistency of the included studies.

### Animals

Only one study examined the presence of farm animals in the domestic environment of households in rural Ethiopia, and found their presence was not associated with stunting [33]. This study did not take into account factors such as the child’s access to the animals, the management of animal feces, or household hygiene practices.

## Other measures of environment and stunting

### Environmental enteropathy

Environmental enteropathy is primarily caused by exposure to environmental pathogens in environments lacking access to WASH services [62]. While not a traditional risk factor, it has been suggested that environmental enteropathy is a causative mechanism for stunting, given that it reduces the absorption of nutrients across the gut barrier, related to the environment in which the child lives [62,63]. Studies that examined the relationship of environmental enteropathy with child growth were therefore included in this review. Testing enteric function using a sugar-based test, one study found that environmental enteropathy was associated with reduced height-for-age Z-score [64]. A second study used the sugar-based test, in addition to rating the cleanliness of a household based on water quality, sanitation, and hand washing. It found children from households rated as clean were more likely to have good enteric function [61]. The impact of geophagy (consumption of dirt, both accidental and deliberate) was studied for both stunting and environmental enteropathy using the sugar-based test [65]. Children who consumed soil were at increased risk of stunting, but there was no association between the test outcome and stunting, suggesting the stunting from geophagy may be mediated by a cause other than environmental enteropathy.

### Intestinal parasites

This review found eight studies that explored the association between parasite infections and stunting. As parasitic infections are context specific, with different environmental and social factors driving the presence of pathogens and human susceptibility to exposure, these reports were considerably heterogeneous in regards to the parasites examined. Half of the included studies (4/8) found that intestinal parasites were associated with stunting. One study associated soil-transmitted helminth infections with an increased risk of child stunting [66], while another found a weak association between the presence of intestinal helminth infection and stunting [67]. This particular study also found no association with protozoal infections and stunting. This is different to the findings by the other studies, which found the presence of the protozoan parasites *G. lamblia* or *E. histolytica* in feces [68], as well as chronic protozoa infections [69], were associated with reduced mean height-for-age Z-score.

Two studies found no association between the presence of one or more helminths in feces and stunting [51,70]. *Trichuris* infection of moderate to high intensity was not associated with stunting for pre-school aged children in Peru [71]. No association was found between the presence of the intestinal parasites *Ascaris lumbricoides, Trichuristrichiura*, or *Giardia lamblia* in stool samples and stunting, although infection with these parasites was significantly associated with dual burden households – those with both stunting children and obese adults [72].

While the reports showed inconsistent results, their association with stunting in some contexts means that parasites should be considered when evaluating communities for stunting prevention.

### Other biological agents

#### Malaria

The impact of malaria infection on stunting was examined in two studies. One of these studies found repeated malarial infections as an infant increased the risk of stunting [73]; however, the other study found no relationship between malarial infection in the child and height-for-age Z-score [21]. The current evidence is insufficient to ascertain the role of malaria in childhood stunting.

#### Mycotoxins

Five papers reported on associations between child exposure to foodborne mycotoxins and stunting. Three of the studies show that higher levels of AF-alb in serum, a biomarker for aflatoxin (a mycotoxin) exposure, was associated with lower height-for-age Z-score [74,75,76]. A similar inverse association was found for height-for-age Z-scores and aflatoxin B1 exposure [77]. One study found that increasing levels of serum AF-alb was associated with lower length-for-age Z scores, that did not reach significance [78]. Two studies reported that fumonisin exposure was inversely related to height-for-age Z-score [78,79]. Two studies found that a longer period of breastfeeding was associated with reduced aflatoxin intake and thus protective against stunting [75,77]. Combined, these studies show a strong association between the consumption of foodborne mycotoxins and stunting.

### Air quality

#### Cooking fuels

The link between children’s growth and exposure to smoke from cooking fuels was explored in seven studies, with both maternal exposure and a child’s direct exposure linked to stunting.

Two studies found child stunting was associated with the use of biomass fuel for cooking [80,81], while another found that a more indirect link with children exposed to unventilated kitchens more likely to be stunted [49]. The effects start in utero, with mothers using biofuel for household’s energy (wood and dung) at an increased risk of delivering small-for-gestational-age infants [82]. A six-month follow up of these babies found that the association continued into childhood, with a 30% increased risk for stunting. Changing to cleaner fuel sources was protective, with two studies finding that cleaner fuels reduced the risk of child stunting, when compared to less clean options [23,83]. One study of the seven we reviewed found no association between biofuel use and child stunting [84]. These studies show a strong association between stunting and the use of biofuels within the home environment.

#### Environmental tobacco smoke

Three studies examined effects of environmental tobacco smoke on child growth. One study examined babies at six months of age and found maternal exposure to environmental tobacco smoke was not associated with stunting [82]. A study of exposure to environmental tobacco smoke, through the presence of males smoking in the domestic residence, found exposure was not related to child stunting [81]. The third study found early growth effects after exposure to environmental tobacco smoke, but these disappeared by two years of age [85]. The evidence from this review would suggest that long-term effects on growth do not occur after early life exposure to environmental tobacco smoke; however, more research is required to be certain.

### Other environmental risk factors

Outside of the traditional environmental health risk factors, such as air and water pollution and other factors already discussed, only a handful of specific risk factors have been studied for their role in child stunting.

#### Pesticides

A study examining children exposed to pesticide use near the home found that the children exposed to pesticides were more likely to be stunted [49]. More research is required to see if this association remains true in different settings.

#### Mercury in seafood

The effect of mercury on children’s growth was examined in two studies. The source of exposure was the consumption of seafood [86,87]. Neither study reported a statistically significant relationship between stunting and mercury, although one did show a non-significant trend between mercury level and reduced height for age Z-score [86].

#### Electromagnetic fields

The effects of electromagnetic fields on fetal growth have been investigated in several studies; however, this review only identified one study on childhood exposure. Children living within 50 m of high-voltage power lines were found to be at risk of stunting [88]. Children who lived close to the power lines were significantly shorter at every year measured up to age 12. Further studies are required to ascertain the true relationship between electromagnetic fields and stunting.

### Casual diagram

**Figure 2:**
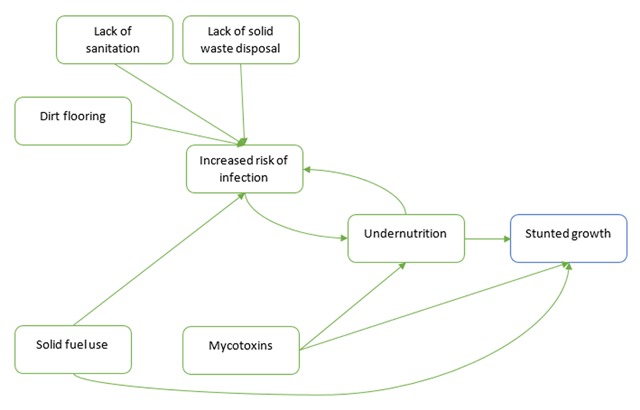
Causal diagram of environmental risk factors and stunting.

The present study identifies five environmental factors as having a strong evidence base to support their association with stunting: lack of sanitation, lack of solid waste disposal, dirt floors, the use of solid fuels in the household, and foodborne mycotoxins. To explore whether these risk factors could potentially have an association with stunting that is independent of nutritional intake, we built a directed casual diagram using the risk factors as exposures and stunting as the outcome. We established there were three broad pathways to stunting: undernutrition, infection leading to undernutrition, and direct effects (Figure 2).

A lack of waste disposal, poor sanitation access, and dirt floors are likely to lead to infectious agents in the environment or increased risk of exposure to those agents. Frequently occurring or chronic infection can cause malnutrition, through increasing caloric needs, catabolism of tissues for energy, sequestering certain micronutrients, and reducing food intake [89,90,91].

Indoor solid fuel use has been associated with an increased risk of respiratory infections, which can be linked to undernutrition; however, we hypothesize that the burning of biomass may be an environmental risk factor with the potential to directly affect growth. There is a dearth of research literature that examines the effect of indoor air pollution on growth, such as skeletal development or growth hormones. From the available research, there is a link between air pollution and lower vitamin D status [92,93,94]. If this association holds true when controlled for UV, it could be hypothesized that children living in homes that burn biomass are exposed to levels of air pollution that may affect their vitamin D status, which has a flow on effect to bone growth. Parathyoid hormone levels are influenced by vitamin D status, and are important hormones for bone growth [95]. Further, indoor air pollution has been linked to a range of inflammatory markers, in both humans and animal models [96,97]. Inflammation affects growth, as proinflammatory cytokines can interfere with hormones involved in regulation of growth hormones and the growth plates of the bone [95].

Mycotoxins are the second environmental risk factor that we identify as having a probable direct effect. Previous studies have explored the mechanisms by which mycotoxins could affect child growth and found that chronic inflammation, interference with the intestinal barrier, and inhibition of protein synthesis occur as a result of mycotoxin ingestion and could interfere with the growth process [98].

## Discussion

This review brings together a list of environmental risk factors that have been studied for their association with childhood stunting. We find that lack of sanitation, lack of waste disposal at the community level, dirt floors in domestic settings, mycotoxins in food, and the burning of solid fuels indoors have sufficient evidence to find an association with childhood stunting. Conversely, the identified literature did not allow for a conclusive finding of some classic environmental risk factors, such as lack of access to clean water. Given that the included papers do not allow us to infer whether association occurs independently of nutritional status, we present a directed causal graph to show that the use of solid fuels and foodborne mycotoxins are environmental risk factors with the potential to directly affect child growth. Future research into environmental risk factors should control for nutritional intake, so that true effect of the environment can be better understood.

Potential limitations of this review is the exclusion of reports not presented in English and the broad search terms, which may have resulted in the unintentional exclusion of studies. The heterogeneous nature of the included studies did not allow for statistical pooling of results. Considerable variation in study design was found between studies in each category. Much of this variation reflects the difficulty of conducting research into environmental health, especially given that exposure data is piecemeal at best. The included studies that examined environmental settings all controlled for poverty markers; however, the specific variables used in each study varied. This reflects the differing markers of poverty in each specific context but makes comparison of confounders more difficult. The contributions – and thus the strength – of this review, is the global focus on childhood stunting and the wide range of environmental risk factors that were captured in the review.

Healthy environmental settings can protect a child’s development, whilst a setting with multiple environmental risk factors puts a child at greater risk of stunted growth [99]. While stunting is slowly declining, the evidence is emerging that global climate change could reverse these gains and lead to increases in stunting. A recent paper exploring the effects of climate variations on child growth, found that changes in temperature and rainfall lead to changes in stunting prevalence [100]. A model built to examine how changes in food availability under climate change would affect stunting, predicts an increase in stunting in the region of 30–50% by 2050 [101]. The relationship between climate, food security, and stunting was examined in a systematic review of 15 papers and found that 80% of the included studies found associations between climate and poor growth [102]. These papers demonstrate that understanding the role of the environment in childhood stunting is vital to protect children now and under a changing climate.

## Conclusion

The results show that a diverse range of environmental risk factors are, to varying degrees, associated with stunting and demonstrates the importance of considering how the environment interacts with nutrition. The environmental risk factors in this study all comprise ‘unhealthy’ child environments. Reducing childhood stunting and maintaining these gains in the face of a changing climate require interventions aimed at reducing stunting to be comprehensive and integrative in their consideration of the role of environmental risk factors.

## Disclosure

The authors have no financial relationships relevant to this article to disclose.
